# Gold Lower Plates

**Published:** 1904-06

**Authors:** L. B. Haskell

**Affiliations:** Chicago.


### GOLD LOWER PLATES.

By Dr. L. B. Haskell, Chicago.

Take an impression always in plaster. Upon removing the model, build up with flaring sides so it will drop readily from the mould. Make Babbitt Metal die. For patterns use tea-chest-lead, Japanese is preferable. Form patterns so as to swage in two pieces, each one extending six and one-half inches beyond the teeth. The plate can be partially formed by lower bending pliers. After swaging each piece separately, swage the two together. Trim the *upper* one the width desired always extending well up *on the necks of the teeth*, as the plate will set steadier and giving more width for strength. Leave the lower section untrimmed so as to have a margin upon which to lay the solder. Borax well the two surfaces, and clamp together with two clamps. Lay the solder all along the margin which was left untrimmed and apply the heat from the upper margin so as to draw the solder between. This will give a nicely fitting plate, reinforced by doubling or lapping the two sections.

It must always be borne in mind that a lower plate, full or partial, must not be so deep on the *lingual* side as to be lifted by the muscles and glands, which often rise above the top of the ridge. When preparing to make a plate always have the patient raise the tongue to the palate and act accordingly.—*Summary.*

